# Sleep status and influencing factors of primary and secondary school teachers in China

**DOI:** 10.3389/fpubh.2025.1661255

**Published:** 2025-09-26

**Authors:** Lian Xue, Ying Chen, Huan Wang, Yin Peng, Yujiao Li, Yi Wang

**Affiliations:** ^1^The Third Hospital of Mianyang (Sichuan Mental Health Center), Sleep Medicine Center, Mianyang, China; ^2^Huaxi MR Research (HMRRC), Department of Radiology, West China Hospital of Sichuan University, Chengdu, China

**Keywords:** primary and secondary school teachers, sleep, anxiety, depression, work stress, influencing factors

## Abstract

**Objective:**

To investigate the sleep status and influencing factors affecting primary and secondary school teachers, this study aims to provide insights for enhancing their sleep quality and establishing effective mechanisms for sleep management and intervention.

**Methods:**

A cross-sectional survey was conducted using cluster sampling to recruit 225 teachers from 58 primary and secondary schools in Mianyang, Sichuan Province, China. Questionnaires included demographic data, Pittsburgh Sleep Quality Index (PSQI), the Dysfunctional Beliefs and Attitudes about Sleep-16 (DBAS-16), Generalized Anxiety Disorder-7 (GAD-7), Patient Health Questionnaire-9 (PHQ-9), and a work stress questionnaire for primary and secondary school teachers. Descriptive statistics were employed to characterize the sleep status, independent sample *t*-tests, Spearman’s correlation analysis, and analysis of variance (ANOVA) were used for univariate analyses of sleep quality, and multiple linear regression was performed to model the influencing factors of sleep quality.

**Results:**

The PSQI score was 8.29 ± 4.58, DBAS-16 score 40.63 ± 12.14, GAD-7 score 7.01 ± 5.21, PHQ-9 score 9.05 ± 6.46, and work stress questionnaire score 67.51 ± 34.04 (standardized to 1.88 ± 0.95). Univariate analysis showed significant differences in sleep quality among teachers with different teaching durations, salary satisfaction, anxiety, depression, work stress, and sleep beliefs/attitudes (all *p* < 0.05). Multiple linear regression analysis identified that teaching duration, anxiety, depression, and work stress were influencing factors of sleep quality (all *p* < 0.05).

**Conclusion:**

The sleep quality of primary and secondary school teachers was generally poor (69.3%), with high detection rates of anxiety (65.3%) and depression (74.7%). Depression (*β* = 0.303, *p* < 0.001) and anxiety (*β* = 0.208, *p* = 0.016) showed strong positive predictive power for sleep quality, indicating that more severe emotional problems were associated with poorer sleep.

## Introduction

Teaching is a meaningful and impactful profession that shapes the development of a nation’s youth (1). In recent years, educational authorities in China have implemented a series of reforms and initiatives aimed at transforming education from traditional knowledge imparting to quality-oriented education. For instance, the General Office of the Communist Party of China Central Committee and the General Office of the State Council jointly issued the *Opinions on Further Reducing the Burden of Homework and Off-Campus Training for Compulsory Education Students* in 2021, which outlines a policy known as the “Double Reduction” (2). The release of the “Opinions” is intended to enhance the primary role of school education, intensify oversight of off-campus training institutions, alleviate parental anxiety, prevent infringements on public interests, establish a secure educational environment, and promote the holistic development and healthy growth of students (3).

As a vital component of professional labor, teaching is regarded as a highly stressful occupation (4, 5). Primary and secondary school teachers, being key drivers in advancing quality-oriented education (6), have their physical and mental well-being under intense societal scrutiny (7, 8). Compared with the general working population, teachers are more prone to insomnia (9, 10). The impact of insomnia on teachers extends beyond personal well-being, affecting work performance and student academic outcomes. Studies have indicated that sleep disorders can lead to cognitive impairments, reduced creativity, and diminished decision-making abilities, underscoring the significance for educators to address sleep issues to achieve optimal performance (11). Insufficient sleep not only poses a risk factor to teachers’ own welfare and health but also, through the process of emotional transmission, transfers the stress caused by sleep deprivation to students (12).

The implementation of such policies has imposed higher demands on teachers, particularly those in primary and secondary schools, leading to significant changes in their work characteristics (13). Teachers are required to extend their working hours to engage in after-school tutoring and student care. This not only elevates the standards for their professional competence but also demands that they assume greater roles and shoulder more responsibilities (14). Against this policy backdrop, have corresponding changes occurred in the sleep and emotional (or psychological) states of teachers, particularly primary and secondary school teachers? Most studies have analyzed the implementation strategies and effects of the “Double Reduction” policy from the perspectives of students and parents, while overlooking the important role and status of teachers as participants in the “Double Reduction” policy. This oversight has led to limitations in certain studies (15). A study based on grounded theory found that in-service primary and secondary school teachers, as well as junior high school teachers, exhibit differences in their understanding of the “Double Reduction” policy, and such differences exert a certain impact on the development of their anxiety levels. Owing to the rapid and complex nature of educational reforms, teachers are prone to experiencing occupational anxiety (16).Another study examined the self-reported occupational burnout levels of primary school teachers in the context of the “Double Reduction” policy and explored the relationship between occupational burnout and depressive symptoms. The study indicated that the rate of occupational burnout among primary school teachers in China is exceptionally high, especially under the background of the “Double Reduction” policy (15). However, there is a dearth of current research on the sleep status of primary and secondary school teachers and its influencing factors. In summary, against the backdrop of the “Double Reduction” policy, exploring the current status of sleep among primary and secondary school teachers and its influencing factors is both innovative and of great necessity. Therefore, this study intends to conduct a preliminary investigation into the current sleep status of primary and secondary school teachers and its influencing factors, so as to provide references for improving the sleep quality of the teacher group and establishing effective sleep management and intervention mechanisms.

## Materials and methods

### Study design and participants

Cluster sampling was employed in this study. A sampling frame was constructed by acquiring the list of 58 primary and secondary schools (32 primary schools and 26 junior high schools) in the urban area of Mianyang City from the Mianyang Municipal Bureau of Education. The primary schools were coded from 1 to 32, while the junior high schools were coded from 1 to 26, respectively. The “RANDBETWEEN” function in Excel was used to randomly generate one number from the primary school codes and one number from the junior high school codes; the primary school and junior high school corresponding to these two numbers were designated as the survey clusters. A full-cluster survey was conducted among teachers in the selected schools from June 2024 to August 2024, and questionnaires were distributed via a combination of online and offline approaches. A total of 228 questionnaires were collected, among which 225 were valid, resulting in a validity rate of 98.7%. The general information collected included gender, age, marital status, educational level, teaching duration, position, professional title, grade taught, and satisfaction with salary, among other variables. This study obtained approval from the Ethics Review Committee of the Third Hospital of Mianyang (Sichuan Mental Health Center). Prior to completing the questionnaire, all participating teachers were informed of the purpose and content of the study, and their informed consent was obtained.

### Measurements

1. *Pittsburgh Sleep Quality Index (PSQI)* (17, 18). It is used to assess sleep quality over a one-month period. A global score and seven component scores can be derived from the scale. The component scores are as follows: subjective sleep quality, sleep latency, sleep duration, sleep efficiency, sleep disturbances, use of sleep medication, and daytime dysfunction. Each component is scored on a range of 0–3, with a total score ranging from 0 to 21; a higher score indicates poorer sleep quality. A global PSQI score greater than 5 indicates poor sleep quality across all age groups (19).
2. *The Dysfunctional Beliefs and Attitudes about Sleep-16 (DBAS-16)*. It is employed to assess dysfunctional cognitions regarding sleep, consisting of 4 factors: consequences of insomnia, worry about sleep, expectations about sleep, and beliefs related to medication use. Fu et al. (20) tested the reliability and validity of the Chinese version of the scale. The Cronbach’s *α* coefficient for the total scale score was 0.786, and the test–retest reliability was 0.928; both values were greater than 0.70, indicating that the Chinese version has good internal consistency and temporal stability. The Chinese version adopts a 5-point Likert scale, with scores ranging from 1 (strongly agree) to 5 (strongly disagree). The total score ranges from 16 to 80, where a lower score indicates a more severe level of irrational beliefs and attitudes toward sleep.
3. *Generalized Anxiety Disorder-7 (GAD-7)* (21, 22). It is used to assess the severity of anxiety symptoms over the past two weeks. The scale are scored on a 4-point Likert scale ranging from 0 (not at all) to 3 (nearly every day), with a total score ranging from 0 to 21. The cutoff scores for mild, moderate, and severe levels are 5, 10, and 15, respectively.
4. *Patient Health Questionnaire-9 (PHQ-9)* (23, 24). This scale comprises 9 items related to depressive disorders, using a 4-point scoring system ranging from 0 to 3. The total score is the sum of the scores of the 9 items, with a total score range of 0 to 27. Cutoff scores of 5, 10, and 20 indicate mild, moderate, and severe levels, respectively.
5. *Work stress questionnaire for primary and secondary school teachers* (25). This scale consists of 36 items, employing a 5-point scoring system with 1 representing “no pressure” and 5 representing “extreme pressure”; a higher score indicates greater pressure.

### Statistical analysis

Statistical analyses were performed using SPSS 22.0. Descriptive statistics were employed to analyze teachers’ demographic characteristics, current status of sleep and emotions, and work stress. Independent samples *t*-test and Spearman correlation analysis were utilized for univariate analysis. Multiple linear regression analysis was applied to establish a model of influencing factors for sleep quality. A *p*-value less than 0.05 was considered statistically significant.

## Results

### Participants’ characteristics

A total of 228 questionnaires were retrieved, among which 225 were valid, yielding an effective response rate of 98.7% (*N* = 225). The demographic profile of the participants is as follows: 186 females (82.7%) and 39 males (17.3%); 70 unmarried individuals (31.1%) and 155 married individuals (68.9%); in terms of age distribution, 82 participants were aged 20–30 years (36.4%), 70 were aged 31–40 years (31.1%), and 73 were over 40 years old (32.4%); regarding educational attainment, 24 had a junior college education (10.7%), 181 held a bachelor’s degree (80.4%), and 20 had postgraduate qualifications (8.9%); by position, there were 121 subject teachers (53.8%), 92 head teachers (40.9%), and 12 administrators (5.3%); in terms of professional titles, 39 had no title (17.3%), 82 held a primary title (36.4%), 82 held an intermediate title (36.4%), and 22 held a senior title (9.8%); concerning the grades taught, 189 taught at the primary level (84.0%) and 36 taught at the junior high level (16.0%); with respect to teaching experience, 68 had 1–5 years of experience (30.2%), 41 had 6–10 years (18.2%), 41 had 11–15 years (18.2%), 17 had 16–20 years (7.6%), and 58 had more than 20 years (25.8%); regarding salary satisfaction, 139 were satisfied (61.8%), 50 had a neutral attitude (22.2%), and 36 were dissatisfied (16.0%) (Table 1).

**Table 1:** Participants’ characteristics (*N* = 225).

Variable	Group	Frequency	Proportion (%)
Gender	Male	39	17.3
	Female	186	82.7
Age (years)	20–30	82	36.4
	31–40	70	31.1
	>40	73	32.4
Marital status	Single	70	31.1
	Married	155	68.9
Educational level	Junior college	24	10.7
	Bachelor’s degree	181	80.4
	postgraduate	20	8.9
Teaching experience	1–5 years	68	30.2
	6–10 years	41	18.2
	11–15 years	41	18.2
	16–20 years	17	7.6
	>20 years	58	25.8
Position	Subject teacher	121	53.8
	Head teacher	92	40.9
	Administrator	12	5.3
Professional title	No title	39	17.3
	Primary title	82	36.4
	Intermediate title	82	36.4
	Senior title	22	9.8
Grades taught	Primary school	189	84.0
	Junior high school	36	16.0
Salary satisfaction	Satisfied	139	61.8
	Neutral	50	22.2
	Dissatisfied	36	16.0

### Scale scores

**Figure 1:**
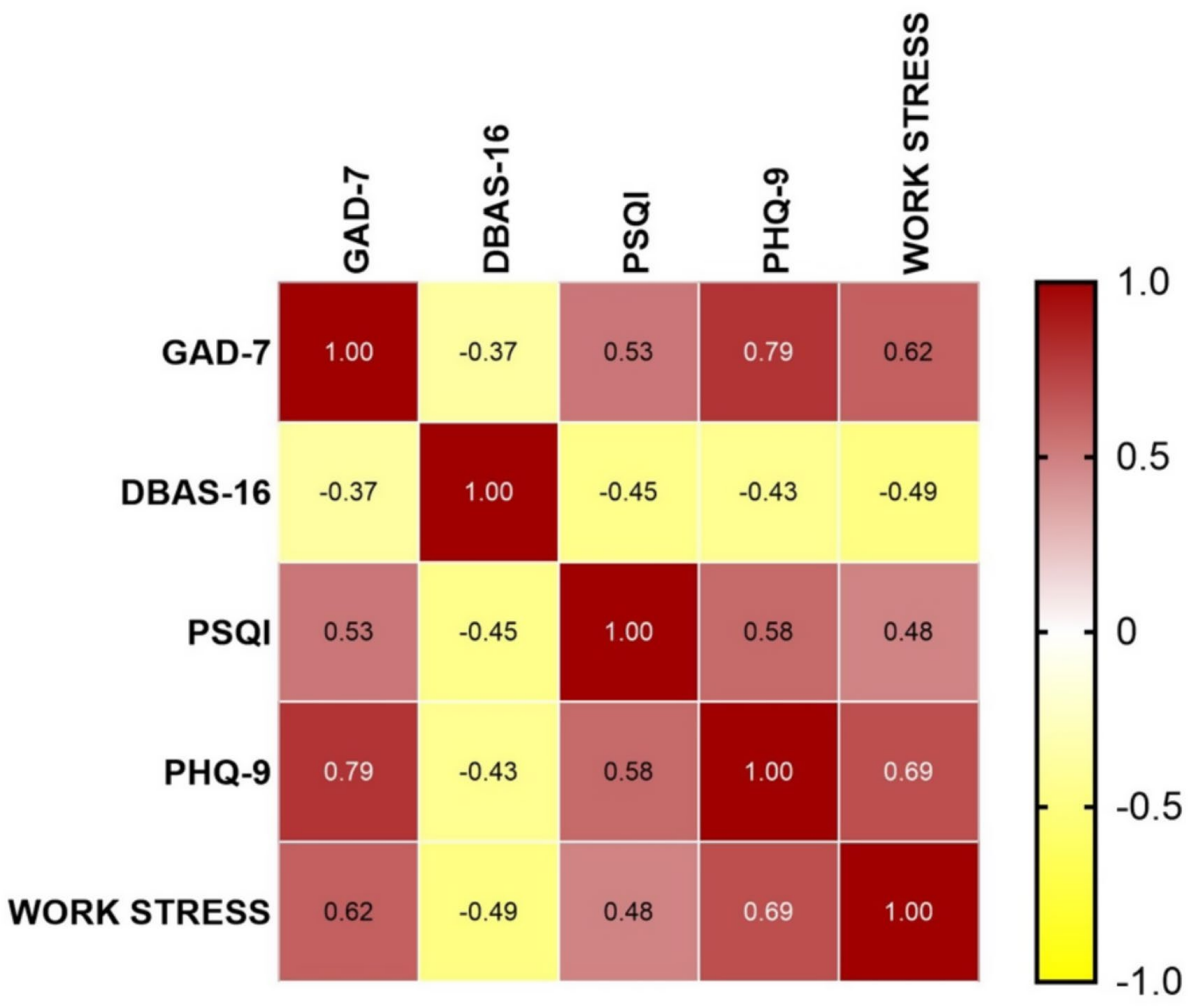
Heatmaps of each scale.

The internal consistency validity (measured by Cronbach’s *α* coefficient) of each scale in this study is presented as follows: PSQI: 0.919; GAD-7: 0.955; PHQ-9: 0.936; Work Stress Questionnaire: 0.946. The PSQI score was (8.29 ± 4.58), with 156 participants (69.3%) scoring above 5. The DBAS-16 score was (40.63 ± 12.14), as detailed in Table 2. The GAD-7 score was (7.01 ± 5.21), and the PHQ-9 score was (9.05 ± 6.46), indicating mild anxiety and mild depressive symptoms, as shown in Table 3. The score of the Primary and Secondary School Teachers’ Work Stress Questionnaire was (67.51 ± 34.04) (standardized score: 1.88 ± 0.95), as presented in Table 4. The heatmaps of each scale are detailed in Figure 1.

**Table 2:** Scores of sleep quality, sleep-related maladaptive beliefs and attitudes among primary and secondary school teachers (*N* = 225).

Item	Mean ± Standard deviation
PSQI	8.29 ± 4.58
DBAS-16	40.63 ± 12.14
Dimensions	
Consequences of insomnia	12.70 ± 4.44
Worry about sleep	14.35 ± 5.47
Expectations about sleep	3.57 ± 1.73
Cognitions about medication	10.00 ± 2.82

**Table 3:** Scores of anxiety and depressive symptoms among primary and secondary school teachers (*N* = 225).

Item	Mean ± Standard deviation	Positive cases	Negative cases
		*N* (%)	*N* (%)
GAD-7	7.01 ± 5.21	147 (65.3)	78 (34.7)
PHQ-9	9.05 ± 6.46	168 (74.7)	57 (25.3)

**Table 4:** Work stress questionnaire for primary and secondary school teachers (*N* = 225).

Item	Mean ± Standard deviation	Standardized (Mean ± Standard deviation)
Total scale	67.51 ± 34.04	1.88 ± 0.95
Educational and teaching reform	3.20 ± 2.38	1.60 ± 1.19
Students	11.88 ± 6.54	1.98 ± 1.09
School management	15.52 ± 8.22	1.93 ± 1.03
Job characteristics	13.16 ± 6.76	2.19 ± 1.13
Career development	5.50 ± 4.01	1.38 ± 1.00
Physical and mental characteristics	6.94 ± 4.22	1.74 ± 1.05
Family	3.47 ± 2.55	1.73 ± 1.28
Society	7.84 ± 4.46	1.96 ± 1.12

### Univariate analysis of sleep quality among primary and secondary school teacher

Univariate analysis was conducted with the total score of the PSQI as the dependent variable. The results showed that there were significant differences in sleep quality among primary and secondary school teachers with different teaching durations, salary satisfaction levels, anxiety emotions, depressive emotions, work stress, and sleep beliefs and attitudes (all *p* < 0.05) (Table 5).

**Table 5:** Univariate analysis of sleep quality among primary and secondary school teacher (*N* = 225).

Variable	Category	Sleep quality score ( $\bar{x}$ ±*s*)	Statistic value	*p*-value^a^
Gender	Male	7.67 ± 3.95	−0.941	0.348
	Female	8.42 ± 4.69		
Age (years)	20–30	8.09 ± 4.42	2.169	0.117
	31–40	7.63 ± 3.47		
	>40	9.16 ± 5.51		
Marital status	Single	8.13 ± 5.25	−0.362	0.717
	Married	8.37 ± 4.25		
Educational level	Junior college	10.08 ± 7.52	2.202	0.113
	Bachelor’s degree	8.13 ± 4.07		
	postgraduate	7.60 ± 4.03		
Teaching experience	1–5 years	7.24 ± 3.68	3.946	0.004
	6–10 years	9.56 ± 4.48		
	11–15 years	6.88 ± 3.68		
	16–20 years	8.71 ± 3.69		
	>20 years	9.52 ± 5.78		
Position	Subject teacher	8.59 ± 4.64	0.943	0.391
	Head teacher	8.10 ± 4.63		
	Administrator	6.83 ± 3.21		
Professional title	No title	7.10 ± 4.13	1.614	0.187
	Primary title	8.23 ± 4.08		
	Intermediate title	9.01 ± 5.39		
	Senior title	7.95 ± 3.37		
Grades taught	Primary school	8.36 ± 4.52	0.498	0.619
	Junior high school	7.94 ± 4.93		
Salary satisfaction	Satisfied	7.64 ± 4.83	4.410	0.013
	Neutral	8.90 ± 4.12		
	Dissatisfied	9.97 ± 3.64		
GAD-7			0.620	<0.001
PHQ-9			0.678	<0.001
Work stress questionnaire			0.545	<0.001
Educational and teaching reform			0.457	<0.001
Students			0.452	<0.001
School management			0.547	<0.001
Job characteristics			0.698	<0.001
Career development			0.491	<0.001
Physical and mental characteristics			0.710	<0.001
Family			0.544	<0.001
Society			0.566	<0.001
DBAS-16			−0.552	<0.001
Dimensions			−0.470	<0.001
Consequences of insomnia			−0.589	<0.001
Worry about sleep			−0.213	0.001
Expectations about sleep			−0.275	<0.001
Cognitions about medication				

A *p*-value indicates differences in general data regarding sleep quality. The relationships between continuous variables and sleep quality were analyzed using Spearman or Pearson correlation analysis; the relationships between categorical variables and sleep quality were analyzed using *t*-test or one-way analysis of variance (ANOVA).

### Multivariate analysis of sleep quality among primary and secondary school teachers

**Figure 2:**
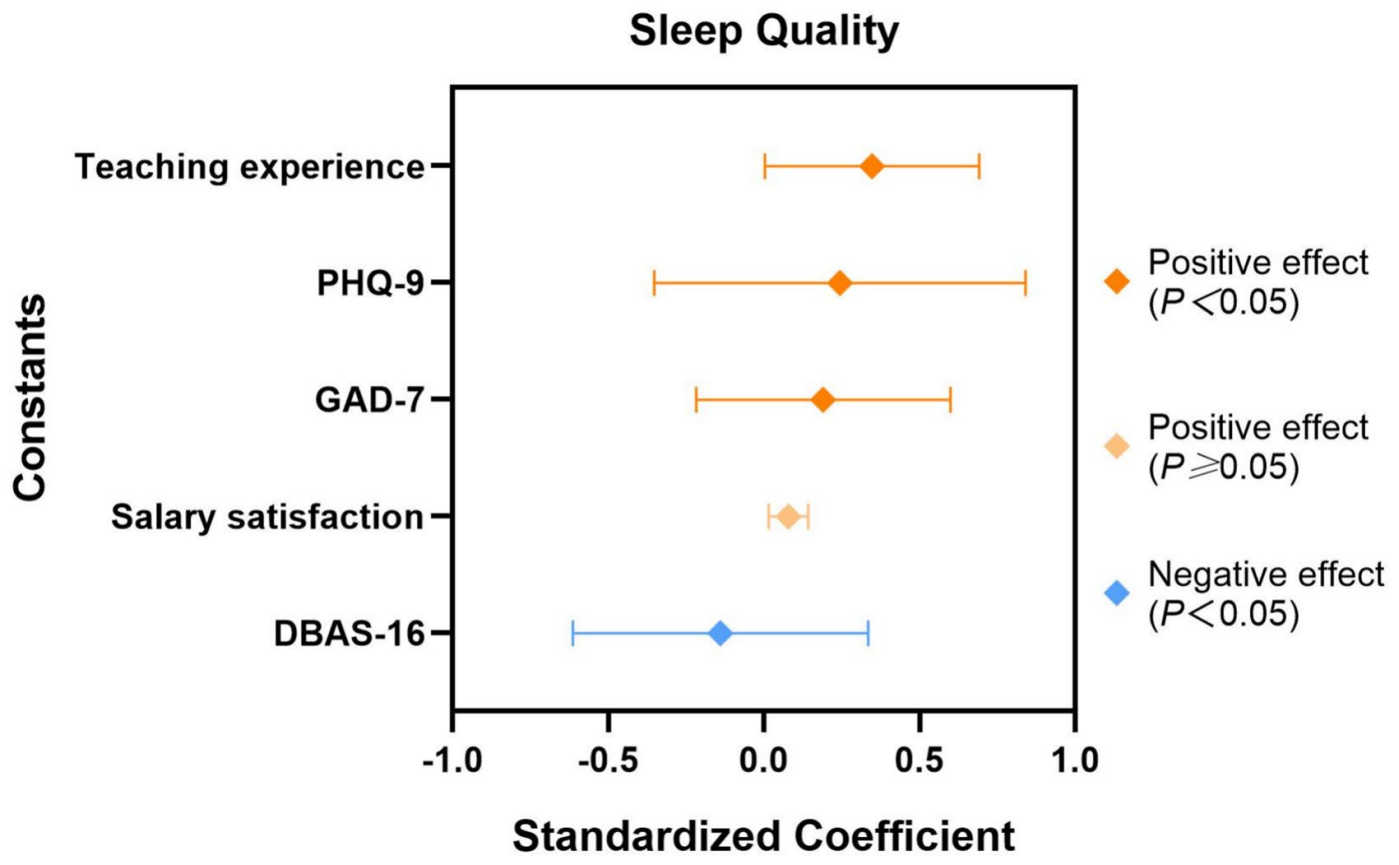
Forest plot of regression effects.

Taking teachers’ sleep quality as the dependent variable and the variables with statistically significant results in the univariate analysis as independent variables, a multivariate linear regression was applied for multivariate analysis. The results showed that the influencing factors of sleep quality among primary and secondary school teachers included teaching duration, anxiety, depression, and work stress (all p < 0.05). See Table 6 for details. The forest plot was generated using the standardized coefficients from the regression equation and their corresponding 95% confidence intervals, as detailed in Figure 2.

**Table 6:** Multiple linear regression analysis of sleep quality among primary and secondary school teacher (*N* = 225).

Variable	Regression coefficient	Standard Error	*β*	95% Cl	*t*-value	*p*-value
Constant	7.293	1.317		(4.698–9.888)	5.539	<0.001
Teaching experience	0.445	0.153	0.153	(0.143–0.747)	2.903	0.004
Salary satisfaction	0.110	0.332	0.018	(−0.545–0.765)	0.332	0.740
GAD-7	0.183	0.075	0.208	(0.035–0.331)	2.430	0.016
PHQ-9	0.215	0.063	0.303	(0.091–0.339)	3.422	<0.001
Work stress questionnaire	−0.090	0.022	−0.238	(−0.132–0.047)	−4.160	<0.001

## Discussion

This study preliminarily explores the current status of sleep among primary and secondary school teachers and its influencing factors. Through a survey of 225 primary and secondary school teachers in Mianyang, Sichuan Province, it reveals the prevalent sleep problems in this group and their underlying causes.

### Current status of sleep, anxiety, depression, and work stress among primary and secondary school teachers

Previous studies have indicated that a PSQI score higher than 5 indicates poor sleep quality across all age groups (19). Approximately 30 to 50% of adults experience occasional sleep difficulties, and 6 to 13% meet the diagnostic criteria for insomnia disorder (26, 27). The proportion of teachers with poor sleep quality ranges from 55 to 61% (9, 28, 29). Among Chinese teachers, the prevalence rates of sleep disorders and sleep deprivation are 20.0 and 31.7%, respectively (30) In the present study, the average PSQI score of primary and secondary school teachers was 8.29 ± 4.58, with 156 teachers (69.3%) scoring higher than 5. These figures are higher than the reported rates of poor sleep quality in both the general adult population and teacher groups in previous studies. In this study, the total score of the DBAS-16 was 40.63 ± 12.14, suggesting that this group generally has poor sleep quality and harbors sleep-related dysfunctional cognitions. The total score of DBAS-16 and all its dimensions were significantly negatively correlated with the PSQI score (*p* < 0.001), among which “worry about sleep” (*r* = −0.589) and “consequences of insomnia” (*r* = −0.470) showed the strongest associations. This may be related to the “cognitive-arousal” model, whereby excessive worry about sleep and catastrophic interpretation can directly activate the physiological arousal system (e.g., increased heart rate, rumination), thereby inhibiting the ability to initiate and maintain sleep (31, 32) Primary and secondary school teachers not only face objective declines in sleep quality but also are affected by sleep-related dysfunctional cognitions, which exacerbate insomnia. This further highlights the importance of Cognitive Behavioral Therapy for Insomnia (CBT-I), emphasizing the need to specifically correct core beliefs such as “sleep worry” and “catastrophizing consequences” to break the cognitive patterns that maintain insomnia and thereby achieve the goal of improving insomnia.

In addition, the results of this study also indicate that the mean score of anxiety symptoms (measured by GAD-7) among primary and secondary school teachers was 7.01 ± 5.21, reaching the criteria for mild anxiety (score ≥5), with a positive detection rate as high as 65.3% (147 teachers). The mean score of depressive symptoms (measured by PHQ-9) was 9.05 ± 6.46, reaching the threshold for mild depression (5 points), and 74.7% of teachers (168 teachers) met the positive criteria for depression (score ≥5). Previous studies have shown that in terms of anxiety, the prevalence rate among Egyptian teachers (67.5%) is 14 times higher than that in the general population (4.75%), and the prevalence rate of depression is 8 times higher than that in the general population (2.7%) (23.2%) (33). A study conducted in Hong Kong, China, showed that the proportion of teachers suffering from anxiety and depression is 2–3 times that of the general population. The prevalence rates of anxiety and depression among primary and secondary school teachers in Sichuan, Jiangxi, and Shandong provinces of China were 26.44 and 20.41%, respectively, (34), and the prevalence rates of anxiety and depression among primary and secondary school teachers in Dongguan City, Guangdong Province, China were 19.3 and 34.7%, respectively, (35). Although the reported prevalence data vary, they consistently exceed the national standards for Chinese adults (12-month prevalence: 5.0% for anxiety disorders and 3.6% for depression) (36), highlighting a noteworthy issue regarding the mental health of primary and secondary school teachers in China. In this study, the detection rates of anxiety and depression among primary and secondary school teachers in Mianyang City, Sichuan Province, were higher than those reported in the above studies. A plausible reason for this discrepancy is the intense educational competition in Mianyang: as a major educational province in China, Sichuan has Mianyang occupying a leading position in terms of educational quality within the province. The core characteristics of Mianyang’s educational system include the concentration of high-quality schools, a clear orientation toward academic progression, and fierce regional educational competition, which corresponds to the workload characteristics of local teachers—heavy explicit tasks (such as formal teaching, homework correction, and lesson preparation) and a large volume of implicit affairs (including administrative work, teacher training, and home-school communication). However, maintaining such a leading position in education is not an easy endeavor; teachers in this region may need to invest more energy in teaching-related work. Additionally, the local society of Mianyang attaches great importance to “high-quality education,” and the phenomenon of “parents’ comparison over their children’s academic progression” is prevalent, which is indirectly transmitted to the teaching workforce and may further exacerbate teachers’ anxiety and depressive symptoms. These results suggest that in future research and policy formulation, appropriate attention should be paid to regional-specific issues in education to address the mental health challenges faced by teachers in highly competitive educational contexts.

In addition, in the present study, the standardized total score of the Work Stress Questionnaire for primary and secondary school teachers was 1.88 ± 0.95, with the most prominent stressors being “job characteristics” (standardized score: 2.19 ± 1.13) and “students” (1.98 ± 1.09). The former reflects the predicaments faced by teachers, such as heavy workload, numerous trivial and complicated tasks, excessive number of students in a class, and the multiple roles of teachers; the latter directly points to core teaching challenges, including students’ poor learning attitudes, low listening efficiency, significant individual differences, disciplinary violations, and non-compliance with management (37). In contrast, the “career development” stress was the lowest (1.38 ± 1.00), suggesting that teachers are more concerned about the current workload rather than promotion opportunities. In a meta-analysis, Montgomery and Rupp examined the causes and impacts of teacher stress in detail and concluded that stress is associated with emotional responses in this population, such as the experience of stress inducing distress, anxiety, and depression. A cross-sectional study on university teachers showed that two-thirds of teachers experienced work stress at least 50% of the time, and workload was one of the most common sources of stress (38). A study evaluating the psychological states across 26 occupations confirmed this, finding that teaching is among the most stressful occupations (33, 39). In China, with the advancement of educational reforms, significant changes have occurred in the work characteristics of teachers, leading to high stress levels, which in turn trigger anxiety and depression. Anxiety and depression often coexist, and stress is an important factor contributing to the increase in anxiety and depression among teachers (40).

### Influencing factors of sleep among primary and secondary school teachers

A further analysis of the influencing factors of sleep quality revealed that univariate analysis indicated a significant correlation between teaching duration, salary satisfaction, anxiety, depression, work stress, DBAS-16, and PSQI scores. However, after controlling for the interactions among variables through multiple linear regression, the impact of salary satisfaction on sleep was no longer significant, with the core influencing factors focusing on teaching duration, anxiety, depression, and work stress.

In the univariate analysis, both depression and anxiety were significantly correlated with sleep quality. Anxiety (*β* = 0.208, *p* = 0.016) and depression (*β* = 0.303, *p* < 0.001) showed strong positive predictive power for sleep quality, indicating that the more severe the emotional distress, the poorer the sleep quality. This result is mutually corroborated by the scale scores: the mean scores of GAD-7 and PHQ-9 reached 7.01 ± 5.21 and 9.05 ± 6.46, respectively, with the positive detection rates of anxiety and depression as high as 65.3 and 74.7%, highlighting the close interweaving of emotional health and sleep problems. This is consistent with previous studies on teacher populations in Malaysia and Brazil (28, 41). However, in the Malaysian population, after adjusting for confounding factors, the associations between depression, anxiety and sleep quality became non-significant; in contrast, in the present study, depression and anxiety remained strong influencing factors of sleep quality after adjusting for confounding factors.

In both univariate and multivariate analyses, work stress remained a significant factor associated with poor sleep quality, which is consistent with the study by Musa, N. A. et al. (28). Notably, the univariate analysis indicated a positive correlation between work stress and PSQI scores (*r* = 0.545), whereas in the regression model, the total work stress scale showed a significant negative association with sleep quality (*β* = −0.238, *p* < 0.001). This seemingly contradictory result—contrary to the common expectation that higher work stress leads to poorer sleep—may stem from the fact that the standardized stress scores (1.88 ± 0.95) in this study were already at a relatively high level, coupled with the existence of complex mediating or moderating mechanisms in stress perception. This result requires further in-depth investigation in future studies.

Compared with the general population, teachers face greater occupational stress (6, 42), and are prone to more severe psychological problems (43). A large body of research has demonstrated that stress is closely linked to the development of mental illnesses, and chronic stress can trigger or exacerbate mental illnesses (44–46). There are numerous objective factors contributing to this situation (47, 48), such as backward teaching conditions, high teaching requirements imposed by new educational reforms, and poor communication with parents. On the other hand, the sharp increase in the incidence of mental health problems among adolescents has also brought greater pressure to teachers, including students’ bullying, truancy, and even suicidal behaviors. Teachers’ involvement in handling these issues can also affect their mental health (49). Cropley et al. (50, 51) investigated work stress, work reflection, and sleep among school teachers and found that teachers with high work stress had poorer sleep compared with the general population, suggesting that work-related factors have a significant impact on teachers’ sleep quality. Gluschkoff et al. (52) studied psychological stress, social work environment, and depressive symptoms among primary school teachers. The results showed that, compared with other occupations, teachers not only experienced higher levels of work stress but also exhibited more symptoms of poor mental health and insufficient sleep.

In summary, this study found that primary and secondary school teachers are confronted with severe sleep quality problems, whose roots lie not in a single factor but in the combined effects of career challenges accumulated over teaching years, prevalent anxiety and depression, high-intensity work stress, and entrenched dysfunctional sleep beliefs. In particular, depressive emotions and the stressors of “job characteristics” and “students” within work stress constitute core risk factors that impair sleep. This result suggests that improving the sleep quality of primary and secondary school teachers requires comprehensive strategies: further reducing the stress from job characteristics and establishing targeted emotional management and sleep hygiene intervention systems. Online cognitive-behavioral sleep training interventions for teachers (including mindfulness training) have been proven effective in improving post-work sleep quality (53), and emotional labor in teaching (mainly including emotional dissonance caused by expressing positive emotions while hiding negative ones) is another key focus of intervention (54, 55). Only through multi-dimensional interventions can the sleep health level of primary and secondary school teachers be effectively improved, thereby ensuring their physical and mental health as well as the quality of education and teaching.

## Conclusion

This study explored the current status of sleep among primary and secondary school teachers and its influencing factors. Through a survey of 225 primary and secondary school teachers in Mianyang, Sichuan Province, it revealed the prevalent sleep problems in this group and their underlying causes. Primary and secondary school teachers generally have poor sleep quality (69.3%), with high detection rates of anxiety and depression (65.3 and 74.7%, respectively). Depressive emotions (*β* = 0.303, *p* < 0.001) and anxiety (*β* = 0.208, *p* = 0.016) show strong positive predictive power for sleep quality, meaning that the more severe the emotional problems, the poorer the sleep quality. Work stress may indirectly affect sleep quality through emotional issues.

### Study limitation

1. Limitation of cross-sectional study design: This study adopted a cross-sectional survey design, which can only reveal the correlation between sleep quality and various influencing factors among primary and secondary school teachers, but cannot infer the causal relationship between variables.
2. Self-report bias: All scales in this study relied on teachers’ self-reports, which are susceptible to subjective cognitive biases (e.g., social desirability bias, where teachers may conceal or downplay negative emotions and sleep problems). This may lead to situations where the actual detection rates of poor sleep quality, anxiety, and depression are higher than the reported values, or the work stress scores are underestimated, thereby affecting the objectivity and accuracy of the results.
3. School-level selection bias: Although cluster sampling was used in the sampling process—randomly selecting 1 primary school and 1 junior high school from 58 primary and secondary schools in Mianyang City as the survey clusters—the sample only covered 2 schools. Moreover, the heterogeneity of school-level characteristics such as school type (e.g., public/private), school scale, and teaching quality ranking was not considered.
4. Failure to consider the nested structure of data: Teacher data have an inherent nested attribute. Teachers in the same school or class may show clustering in indicators such as sleep quality and work stress due to common factors like teaching environment, management mode, and work atmosphere. This study may have ignored the potential impact of school/class-level variables (e.g., school management intensity, number of students in a class), leading to bias in the effect estimation of individual-level influencing factors and affecting the model fit and result reliability.
5. Inadequate control of potential confounding factors: This study did not collect information on whether teachers had chronic diseases (e.g., hypertension, thyroid dysfunction, and other physical diseases that may affect sleep), a history of mental illnesses, or whether they took sedative-hypnotic drugs, anti-anxiety/antidepressant drugs, etc. This may interfere with the true correlation between emotions, work stress, and sleep quality. In addition, this study did not incorporate basic demographic variables such as age and gender into the regression model for confounding adjustment. Although the univariate analysis revealed no significant differences in sleep quality among teachers of different ages or genders (all *p* > 0.05), these variables, as classical basic confounding factors, may confound the effect estimation through potential multicollinearity with other independent variables.
6. Risk of common method bias: All variables in the study (including independent variables and dependent variables) were collected through the same set of questionnaires, and completed by the same respondents (teachers) at the same time point, resulting in the possibility of common method bias.

Future studies will further avoid the aforementioned limitations, adopt multi-center and multi-level sampling designs, introduce objective measurement methods, and develop targeted intervention programs based on teachers’ career stages.

## Data Availability

The original contributions presented in the study are included in the article/supplementary material, further inquiries can be directed to the corresponding author.
